# Food insecurity and health outcome nexus: empirical evidence from the informal sector enterprises in Bangladesh

**DOI:** 10.1186/s12889-023-15655-2

**Published:** 2023-04-20

**Authors:** Nahid Sultana, Mohammad Mafizur Rahman, Rasheda Khanam, Istihak Rayhan, Roni Hossain

**Affiliations:** 1 School of Business, University of Southern Queensland, Toowoomba, Australia; 2 Department of Economics, Jahangirnagar University, Savar, Bangladesh

**Keywords:** Food insecurity, Self-assessed health status, Informal workers, COVID-19, I18, I31, J46

## Abstract

**Background:**

Food insecurity indicates the difficulty of constantly obtaining adequate food because of limited economic resources. Food insecurity challenges the desired health outcomes. Although extensive literature has examined the associations between food security and health, low-wage informal sector workers have been less frequently addressed in this topic. The present study has focused on food insecurity among the workers working in the informal sector enterprises who experienced entrenched disadvantage during COVID-19 and examines the relationship between food insecurity and health status as measured by self-reported physical and mental health conditions.

**Methods:**

This study has utilized cross-sectional data collected from workers working in informal manufacturing and business enterprises in Dhaka city of Bangladesh. The Food Insecurity Experience Scale (FIES) with eight items is used to screen for food insecurity, and the Short Form 12v2 (SF12v2) scale with 12 questions, and validated for use with Bengali respondents, is used to measure the health status of the informal workers. A health production function has been constructed where the health status (both physical and mental) of workers is associated with food insecurity and other socio-economic and health care factors. Empirical analyses of the study have included descriptive statistics, mean score comparisons, and multivariate regression analyses to identify the predictive factors of the physical and mental health status of the workers.

**Results:**

A moderate to severe food insecurity is found to be responsible for the poor health status (both physical and mental) of the selected working group population. Moreover, age over 40 years, having a large family, dissatisfaction with the work place, and the prevalence of occupational health risks are linked to lower physical health, while dissatisfaction with the work place and the incidence of severe diseases contribute to poor mental health status along with food insecurity.

**Conclusions:**

Extending social and economic protection towards health coverage and basic consumption is suggested as an immediate action to save lives and ensure productivity of the informal workers. Besides, an increase in income and ensuring decent working conditions are also recommended for the health, safety and satisfaction of workers working in informal sector enterprises.

## Background

Food insecurity has been recognized as an important social determinant of health [45, 53]. It occurs due to a lack of adequate economic, social, and physical access by people to sufficient, safe, and nutritious food that can satisfy their dietary needs and food preferences for a healthy and active life [22, 43]). It often leads to cycles of fasting and stress, resulting in nutrient deficiencies and weight loss [59]. Food insecurity has significant short-term as well as long-term impacts on physical health and socio-economic participation [13]. It is mostly faced by underprivileged communities, and it affects their health and wellbeing, making them increasingly vulnerable to the risks of starvation, malnutrition, chronic diseases, poor mental health, social conflicts, and inequalities in socio-economic life with an increased probability of hindering the overall developmental activities [13, 40, 69]. Hence, food insecurity has been identified as a multifaceted phenomenon by the United Nations Food and Agriculture Organization (FAO, 2021), which needs to be investigated in light of its complicated impacts. The present study has contributed to this by examining the link between food insecurity and the health status of a susceptible low-income working community operating in the informal sector of Bangladesh.

The informal sector has been accepted globally as a perpetual and significant aspect of urban life [4, 47]. However, the unregulated and unregistered characteristics of informality, precarious working conditions, lower wages, and the involvement of informal enterprises in labor market turnover are suggested as detrimental to health [5, 48, 62]. Informal enterprises are frequently associated with vulnerability and poverty due to the consistent presence of food insecurity and financial crisis [29, 32]. Employment in the informal sector faces a lack of economic security due to irregularities in income, low productivity, health and safety issues, abuse, and exploitation [52]. Workers in informal manufacturing enterprises are exposed to airborne particles, hazardous chemicals, and perilous machinery that lead to a higher prevalence of respiratory diseases [17, 28]. Therefore, informal employment is considered a social determinant of health and embodies a serious problem for public health in terms of poor physical and mental outcomes [48, 62].

The outbreak of the COVID-19 pandemic put 3.3 billion global workforces at risk of losing their livelihoods [33], and it was severe for the informal sector that encompassed a large share of employment in developing countries [5, 17, 77]. Workers in the informal sector were one of the most affected groups in the COVID-19 context, recounting their inability to afford food for sustenance [4, 26]. Informal sector enterprises do not have access to government support programs or formal bank loans [30, 33] and in extreme cases, these enterprises were forced to close their business and production units either momentarily or permanently, which led to job losses and a surge in poverty [30, 33]. Therefore, the informal sector workers were more likely to suffer severe income losses [9, 55, 65]. Since they have limited or no buffers such as savings or access to government support programs, many of them were unable to feed themselves and their families after losing their means to earn an income in the COVID-19 context [55]. This impacted their livelihood and undermined their food security [30, 33]. Food insecurity exacerbated health conditions for these workers with financial challenges due to the known trade-off between purchasing food and meeting health care needs [45]. Workers in this sector are susceptible to a lack of social protection and have poor access to public health care [77]. To this group of people, no income means no food or, at best, less food and less nutritious food [33, 77]. They live with this uncertainty throughout the years, which takes a toll on their health and is one of the worst consequences they have faced during the COVID-19 years.

In the pandemic period, public health issues and their impact on urban informal enterprises became critical, particularly for cities with poor public health systems, the undocumented presence of rural migrants, and the dominance of informal settlements [54]. The financial collapse, permanent closure of many medium and small manufacturing enterprises (MSMEs) and formal absence of income replacement pushed numerous people to resort to the informal sector by making a living as microbusiness owners, own-account workers or service providers, mostly in low and lower-middle-income countries, which often suffer from weak social protection systems and low social coverage. Some formal MSMEs were also pushed into informality [33]. Under such a context, the International Labor Organization (ILO) called for policies to provide income, food support, and adequate health support to the poor and vulnerable informal workers [33]. By conducting an investigation on the workers of the informal sector, this study expects to contribute to policy measures with the suggestions based on empirical evidence. This investigation on urban informal sector workers will also contribute to the effort of diminishing hunger and food insecurity by identifying a food insecure sub-population, while identification of such groups through their demographic characteristics and geographic location is felt necessary for the achievement of the Sustainable Development Goals (see [21]).

In developing countries like Bangladesh, economic lockdown measures carry a serious trade-off in terms of economic welfare, poor nutrition, and hunger [3, 60]. This study, which has investigated the nexus between food insecurity and the health outcomes of workers in the informal sector, was conducted in Bangladesh for some reasonable facts. First, 99% of industrial units in Bangladesh are either micro, small, or medium-sized enterprises, and a large number of those are informally operated [2]. Informal jobs dominate in all sectors of the Bangladeshi economy. Among the broad sectors, the agriculture sector contains 95%, and the industry sector contains 90% of jobs classified as informal [30, 35, 52]. According to the ILO, 57% of total employment in Bangladesh is in a delicate category due to a lack of formal contracts [30]. In urban areas of Bangladesh, around 13.1 million jobs are informal [30]. However, the surge of the pandemic has diminished the employment opportunity in this sector and pushed the unskilled informal wage workers into the constraints of their purchasing power [8, 19, 39, 41, 49, 51]. Second, the agricultural production and employment in Bangladesh are vulnerable to a multitude of shocks since it belongs to the group of countries that are most at risk of weather-related hazards in the Asia and Pacific region. Economic and social progress are uneven among income groups and geographic regions of the country. The latest estimate based on the year 2018–2020 suggests that, on average, 31.9% of the population is experiencing moderate or severe food insecurity, while the prevalence of undernourishment is still 9.7%. The COVID-19 pandemic has added adverse consequences to food consumption and nutrition in Bangladesh [20]. Third, in the historical context of poor food security standing of households in Bangladesh, the adverse effects of the pandemic and countrywide lockdown have extended beyond income shocks and affected people’s food security [3]. Bangladesh is ranked 84 out of 113 countries globally and scored the lowest among the South Asian countries as per the Global Food Security Index 2021 [31]. By shedding light on hunger and food insecurity among workers in urban informal settlements of Bangladesh, this study aims to suggest policies after confirming its association with worker health.

## Methods

### Study design and data collection

The present study has to follow a cross-sectional design because the required data on informal sector enterprises and informal sector workers are unavailable in official sources. A cohort (*n* = 267) of workers employed in medium and small informal enterprises registered with local authorities (but not with a national body) and comprised of  labor without any formal contract, was selected for the interviewer-administered survey. The workers are classified as informal depending on their contractual status. The study was conducted in August-December 2021 in urban areas of Dhaka, Bangladesh. A purposive sampling technique was followed for the study, where workers were selected randomly from three types of manufacturing enterprises: leather and leather products, plastic and small machinery, and dyeing, fabrics, and clothing. Informal enterprises under these three categories of industries are infamous for their polluting activities and unhealthy work environments and have been found to be clustered in three areas of Dhaka city in Bangladesh. Hemayetpur area was selected to survey workers informally employed in the leather factories, Lalbugh (including Islambugh) area was selected to survey informally employed workers in firms within the plastic and small machinery industries, and Keraniganj area was selected to survey informal workers in firms within the dying and clothing industries. Workers were randomly sampled from each enterprise, based on the relative proportion of the workers working in the firm and the overall targeted sample size for each industry. The total sample size was 267 (90 from each industry, of which 3 had to be dropped due to incomplete information). A structured questionnaire was developed in English and translated back into the local language (Bengali) for the survey. A paper based survey was conducted face-to-face among the workers, following all the health and safety rules imposed by the COVID-19 pandemic. After signing the consent form, the potential respondents participated in the interviewer-administered survey. Attending surveyors provided assistance to the respondents who had faced difficulty in reading and answering the survey questions due to a lack of education. Each survey session lasted for about 40–45 min and began with the surveyors reading a verbal consent statement explaining the purpose and potential use of the research, under the supervision of research assistants. The cases where surveyors assisted the respondents by reading consent form and survey questions due to their lack of education were recorded, and all the survey information was transferred to the password-protected software (KOBO Toolbox) on the computer after being translated back into the English language. The saved information in software, from which information could be easily converted into usable data format, was then transferred and stored on a secured drive of the University of Southern Queensland (UniSQ), Australia. During the translation and data entry processes, care was taken to maintain the accuracy of the answers and the error-free entry of quantitative data. The completeness and quality of the data input were cross-checked by the research assistants and the researchers.

### Research participants and ethical considerations

The research protocol was thoroughlyreviewed and finally approved by the ethical board of the UniSQ, Australia (UniSQ HREC ID: H21REA014). It followed rigorously all the steps of data collection guided by the National Statement on ethical conduct in human research 2007 (updated 2018) involving human participants. A local team for data collection was constructed, headed by academic members from the Department of Economics of Jahangirnagar University, Dhaka, Bangladesh (also the co-authors of this article), who were completely aware of the ethical norms of data collection involving humans. The team closely monitored the application of the research protocol, which was approved for this study after ensuring its compliance with international standard. During the information sessions, potential participants were provided with consent forms, where the aims and objectives of the project were mentioned, and the interviewers/surveyors explained verbally the objectives, procedures, risks, and benefits of the study. The survey questionnaire was supplied only to the workers who gave their consent to taking part in the survey after having all the information about the project. The interview team obtained both verbal and written consent from the participants, and the verbal consent statement was read to the participants at the beginning of the survey session who were uneducated. Interviews were conducted outside of the enterprise premises (e.g., a tea-stall, a river bank, or an open space). Participation in this study was completely voluntary, and participants had the complete freedom to withdraw from the survey at any time they wanted without any penalties. The participants were also informed that the data would be kept confidential and used only for research purposes by the researchers. A contact number was provided to each participant of the survey so that measures could be taken in case participants needed any post-interview supportive counseling or assistance.

### Empirical strategy

Asking people directly about their experience of food insecurity has been used for more than a decade by many countries as a tool to collect information and monitor food insecurity. This study has followed this technique applicable to the reference country, Bangladesh, to assess food insecurity, while the self-reported health status (both physical and mental) of workers has been used for assessing health outcomes. Following [14, 70] this study has conducted a multivariate analysis where the association between food insecurity and health outcome was investigated after controlling for other socio-demographic, economic, and health related factors.

The health production function for the study can be presented as below:

1 $${H}_{i}=f({F}_{i},{E}_{i},{S}_{i}, {HC}_{i}, {WE}_{i}, {e}_{i})$$

where, *Hi* is the health status (Physical and mental) of workers and is a function of food insecurity ($${F}_{i})$$ and other economic factors (*E*_*i*_), Social factors (*S*_*i*_), health care factors (*HC*_*i*_), environmental factors (*WE*_*i*_), and unobserved factors (*e*_*i*_) for individual *i* who work in informal enterprises. The study includes economic factor monthly expenditure besides food insecurity, $${F}_{i}$$. Various socio-demographic variables like age, gender, marital status, family size, education, and working hours are included to represent social factors, while having any of the major diseases is included to represent health care factors. Finally, satisfaction in the workplace, which reflects the workers happiness about the work environment (about job certainty, work relation, and a decent work environment), and occupational health risks, which reflect the risk of exposure to pollution or the possible risk of facing accidents due to a lack of adoption to health safety measures, are included to represent the working environment factors.

#### Measurement of health status

Taking insight from Wang et al. [75], Wan et al. [74], Blanco-Reina et al*. *[11] this study has used the SF12v2 [76] health survey to measure the health-related quality of life (HRQoL) of informal workers around Dhaka city. This study uses the translated and culturally adapted Bengali version of SF12v2 which proves to be a reliable, acceptable, and valid instrument for measuring health-related quality of life by Islam et al. [34]. The SF12v2 provides a broader picture of health status that includes eight domains: physical functioning, role physical, bodily pain, general health perception, vitality, social functioning, role emotional, and general mental health [76]. The interviewer-administered Bengali SF 12v2 questionnaire contains 12 questions items about health perception on two different subscales: Physical Component Summary (PCS), which assesses physical health perception (about general health, physical functioning, role-physical and bodily pain), and Mental Component Summary (MCS) which evaluates the perception of mental health (about role emotional, mental health, vitality and social functioning). Scores are calculated through Likert’s method of summed ratings. In this method the algebraic total of the item scores is used to calculate a score for each question item [34]. Some of the items’ scores had to be recoded so that they were all scored in the same direction. The raw scores are added together and linearly transformed into a 0–100 scale, with higher scores denoting greater health [34]. In the current investigation, the Cronbach's alpha for SF12v2 was 0.80, indicating that the scale has strong internal consistency.

#### Measurement of food insecurity

Food insecurity that indicates a difficulty in constantly obtaining adequate food because of limited economic resources for food is evaluated by administering the interview with eight questions. FAO [21] has proposed the Food Insecurity Experience Scale Survey Module (FIES-SM), which relies on the yes/no answer of the respondents to eight brief questions regarding their access to adequate food, works as a global reference scale of individual food insecurity. This is a quantitative tool to measure the prevalence of food insecurity (at a moderate to severe level) by statistically analyzing the eight questions that enable estimation of error (confidence intervals around the measures produce) and has been used since 2014 as a survey module [21]. This is also an official tool for measuring access to food within the Sustainable Development Goals (target 2.1), and proves its validity and reliability in socially-backward communities in the context of South Asia [56]. FIES recommends that each country arrive at a categorization of food insecurity (FI) that is meaningful to its context. This study defines the status of food security following FAO [21] and frames the questions accordingly. It provides a raw score-based categorization, while high food insecurity (FI) is categorized with a raw score of 0, marginal FI with a raw score of 1–3, moderate FI with a raw score of 4–6 and severe FI with a raw score of 7–8 [16, 67]. According to the FAO [19] report, mild food insecurity is defined as ‘at times during the year, households reduced the quality, variety, and desirability of their diets due to a lack of resources for food, but the quantity of food intake and normal eating patterns were not substantially disrupted’ and moderate to severe food insecurity is defined as ‘at times during the year, eating patterns of one or more household members were disrupted and food intake reduced because the household lacked money and other resources for food’ [19]. In the present investigation, the Cronbach's alpha for FIES was 0.91, indicating that the scale has strong internal consistency.

#### Variables and categories

Age was classified into four categories: 18–30, 31–40, 41–50, and 51 and older, while 18–30 years was chosen as the reference group for the regression analysis. Gender was divided into male and female, with male being the reference category, and marital status was divided into single and married, with unmarried being the reference category. There are two types of families: those with four or fewer members and those with more than four members, with the first serving as the reference category. Working hours were divided into three categories: less than 8 h, 8 h, and more than 8 h, with less than 8 h being the reference category, and monthly expenditure was divided into three categories: less than BDT 15,000, BDT 15,001–30,000, and more than BDT 30,000, with less than BDT 15,000 being the reference. The participants were asked to rate their level of happiness at work, with the options being feeling happy, feeling somewhat happy, and feeling unhappy. Feeling happy was designated as the reference category. Regarding health issues at work, a dichotomous response was included, with the reference category being none at all. The experience of food insecurity was divided into two categories: food secure or mild food insecure and moderate to severe food insecure, with the first one serving as the reference category. Finally, occurrence of any or more of the major diseases, namely, 1. hypertension, 2. heart disease (including coronary heart disease and other heart condition), 3. stroke, 4. hyperlipidemia, 5. liver disease, 6. diabetes mellitus and other endocrine disease, 7. respiratory disease, 8. urinary and reproductive disease, 9. musculoskeletal disease, 10. gastrointestinal disease, 11. dermal diseases, and 12. dental caries or other dental disease [57], was reported into one of three categories: There is no major disease, one major disease, or multiple major diseases. There is no major disease is set as the reference category. Based on the reference categories, it is expected that aged, female, and married workers with a large family size and lower monthly expenditure, feeling unhappy at work, experiencing moderate to severe food insecurity, facing health risks at work, and having one or more major diseases will have a lower health status than their counterparts.

### Data analysis

Data were coded in Excel and all survey data were doubled-entered, checked for missing values, outliers, normality assumptions before data analysis. Statistical analysis were performed by using econometric software STATA version 15.0. First the descriptive statistics of all the variables, including mean, standard deviation, frequency, and percentages were used to summarize and describe the demographic characteristics of the sample. A multivariate regression analysis was used to identify the predictive factors of PCS and MCS. The level of significance of all statistical tests performed was set at *p* < 0.05 and two-tailed.

## Results

Table 1 presents the details of SF12v2 score values. The mean scores of SF12v2 PCS is 46.89 (SD = 7.80) and MCS is 41.34 (SD = 8.03). While the mean score for general health is 39.78 (SD = 9.87), physical function 47.43 (SD = 10.99), role physical 50.11 (SD = 7.12), bodily pain 43.36 (SD = 8.55), role emotional 46.22 (SD = 8.33), mental health 41.21 (SD = 8.75), vitality 41.34 (SD = 8.03), and social functioning 39.28 (SD = 11.05). The mean scores for general health and social functioning are found lower among the informal workers.

**Table 1:** Mean and standard deviation of SF12V2 scoring

Variables	Mean (Std. Dev.)
***Physical Component Summary (PCS)***	46.89 (7.80)
General Health (GH)	39.78 (9.87)
Physical Functioning (PF)	47.43 (10.99)
Role-Physical (RP)	50.11 (7.12)
Bodily Pain (BP)	43.36 (8.55)
***Mental Component summary(MCS)***	41.34 (8.03)
Role Emotional (RE)	46.22 (8.33)
Mental Health (MH)	41.21 (8.75)
Vitality (VT)	41.34 (8.03)
Social Functioning (SF)	39.28 (11.05)

The baseline characteristics of the study subjects are summarized in Table 2 along with the mean score of SF12v2. Among the 267 participants, 59.55% are in the age group of 18–30 years, 22.10% are in the age group of 31–40 years, 9.74% are in the group of 41–50 years, and 8.61% are in the group older than 51 years. Among the total participants, 89.51% are men; 62.55% are married; 64.79% have more than four family members; 11.61% have no formal education, 55.06% have a primary education, and 30.34% have a secondary education. Additionally, 87.27% of participants work more than eight hours per day; 59.93% have a family income of less than BDT 15,000; and 30.34% have an income between BDT 15,000 and 30,000. About 83.46% of workers have reported that they face health risks at work, 18.73% report having one major disease, 49.06% have multiple major diseases, and 66.29% have moderate to severe food insecurity.

**Table 2:** SF mean score comparisons by socio-demographic and health related variables

**Variables**	**n (%)**	**PCS-12 mean score (Std. Dev.)**	**Prob > F**	**MCS-12 mean score (Std. Dev.)**	**Prob > F**
Total sample	267 (100)				
**Age**					
18–30	159 (59.55)	48.79 (6.29)	< 0.001	41.41 (8.35)	< 0.001
31–40	59 (22.10)	47.19 (7.91)		40.97 (8.10)	
41–50	26 (9.74)	42.71 (7.01)		41.41 (6.98)	
≥ 51	23 (8.61)	37.70 (9.81)		41.76 (7.07)	
**Gender**					
Male	239 (59.51)	47.40 (7.49)	< 0.001	41.51 (8.12)	< 0.001
Female	28 (10.49)	42.49 (9.09)		39.89 (7.14)	
**Marital Status**					
Unmarried	100 (37.45)	49.23 (5.73)	< 0.001	41.27 (8.29)	< 0.001
Married	167 (62.55)	45.49 (8.53)		41.39 (7.89)	
**Family size**					
≤ 4	94 (35.21)	48.10 (7.04)	< 0.001	39.67 (7.56)	< 0.001
> 4	173 (64.79)	46.23 (8.13)		42.25 (8.15)	
**Education**					
No education	31 (11.61)	43.93 (8.33)	< 0.001	42.07 (9.30)	< 0.001
Primary	147 (55.06)	47.73 (7.95)		41.01 (8.42)	
Secondary	81 (30.34)	46.56 (6.89)		41.05 (6.79)	
≥ Higher secondary	8 (3.00)	46.11 (10.04)		47.51 (4.75)	
**Working Hours**					
< 8 h	7 (2.62)	52.17 (2.83)	< 0.001	43.20 (9.88)	< 0.001
8 h	27 (10.11)	46.63 (9.41)		43.95 (9.55)	
> 8 h	233 (87.27)	46.76 (7.67)		10.98 (7.75)	
**Monthly expenditure**					
≤ 15,000 BDT	144 (53.93)	46.20 (7.58)	< 0.001	39.07 (7.17)	< 0.001
15,001–30,000 BDT	81 (30.34)	46.46 (8.87)		41.05 (7.31)	
> 30,000 BDT	42 (15.73)	50.08 (5.38)		49.71 (6.67)	
**How do you feel at your workplace? (Satisfaction with workplace)**					
Happy	27 (10.11)	51.14 (6.22)	< 0.001	49.97 (8.27)	< 0.001
Moderately happy	207 (77.53)	46.97 (7.39)		40.60 (7.54)	
Not happy	33 (12.36)	42.92 (9.56)		38.96 (6.44)	
**Health risk at workplace**					
No	222 (83.46)	47.71 (6.90)	< 0.001	41.95 (8.38)	< 0.001
Yes	45 (16.54)	42.66 (10.48)		38.41 (5.10)	
**Major Diseases**					
No major disease	86 (32.21)	48.56 (6.63)	< 0.001	47.59 (8.47)	< 0.001
One major disease	50 (18.73)	46.65 (6.42)		41.20 (5.33)	
More than one	131 (49.06)	45.88 (8.80)		37.30 (5.65)	
**Food insecurity**					
Food secure or mild	90 (33.71)	51.29 (4.82)	< 0.001	46.39 (8.91)	< 0.001
Moderate to Severe	177 (66.29)	44.65 (8.08)		38.77 (6.13)	

In Table 2, findings with both PCS and MCS have revealed the significance of all variables considered in the model, i.e., age, gender, marital status, family size, education, working hours, monthly expenditure, satisfaction in the workplace, occupational health issues, major diseases, and food insecurity all are related to the physical and mental health of the sample.

The outcomes of the estimation of multivariate linear regression for physical health components are presented in Table 3. When compared to workers in the reference group of 18 to 30 years, workers who are 41 to 50 years old have a mean score that is 3.5989 [SD = 1.56; 95% CI = -3.79 to 0.80] units lower, and workers over 51 years have a mean score that is 8.44 [SD = 1.65; 95% CI = -11.69 to -5.20] units lower. This leads to the conclusion that for the selected workers working in the informal manufacturing sector, the mean score for physical health declines with age. Besides, when controlling for all other variables, it is revealed that female workers typically have a mean score of physical health that is 4.8686 [SD = 1.35; 95% CI = -7.52 to -2.21] units lower than that of male workers. Furthermore, the family size variable's slope coefficient was found to be negative and significant at the 5% level of significance. According to the estimates, families with more than four members tend to have a mean score of physical health that is 2.2584 [SD = 0.89; 95% CI = -4.00 to -0.51] units lower than families with four or fewer members.

**Table 3:** Result of Multivariate linear regression for physical health components (PCS-12)

Variables	Coefficient	Std. Err	*p*-value	95% CI	
**Age**	Ref. 18–30 Years				
31–40	-1.4942	1.1635	0.200	-3.7859	0.7976
41–50	-3.5989^b^	1.5554	0.022	-6.6624	-0.5353
≥ 51	-8.4421^a^	1.6463	< 0.001	-11.6848	-5.1993
**Gender**	Ref. Male				
Female	-4.8686 ^b^	1.3484	< 0.001	-7.5245	-2.2127
**Marital Status**	Ref. Unmarried				
Married	-1.6768	1.0680	0.118	-3.7804	0.4268
**Education**	Ref. No education				
Primary	0.8027	1.3078	0.540	-1.7732	3.3786
Secondary	-0.9959	1.3889	0.474	-3.7317	1.7397
≥ Higher secondary	-1.6688	2.5729	0.517	-6.7364	3.3988
**Family Size**	Ref. ≤ 4				
> 4	-2.2584 ^b^	0.8868	0.011	-4.0051	-0.5117
**Working hours**	Ref. < 8 h				
8 h	-3.7015	2.6990	0.171	-9.0176	1.6146
> 8 h	-2.4721	2.4496	0.314	-7.2969	2.3527
**Monthly expenditure**	Ref. ≤ 15,000 BDT				
15,001–30,000	0.9306	0.9556	0.331	-2.8128	0.9517
> 30,000	-1.5936	1.4733	0.280	-4.4955	1.3083
**Moderate to severe food insecurity**	Ref. No				
Yes	-6.0370 ^a^	1.0934	< 0.001	-8.1907	-3.8834
**Workplace Satisfaction**	Ref. Happy				
Somewhat happy	-2.3423^c^	1.1576	0.094	-5.0857	0.4012
Not happy	-3.6209 ^b^	1.0180	0.041	-7.0981	-0.1436
**Health risk at workplace**	Ref. No				
Yes	-2.7282 ^b^	1.1537	0.019	-5.0007	-0.4558
**Major Diseases**	Ref. No				
One	-0.7703	1.1576	0.506	-3.0503	1.5098
More than one	1.2314	1.0180	0.228	-0.7737	3.2367
***Diagnostic check***					
R-Square	0.4103				
RMSE	6.2305				
F-Stat	9.0076 (*p*-value < 0.001)				

^a^ represents significance at 1% level, ^b^ represents significance at 5% level and ^c^ represents significance at 10% level

When all other variables are taken into account, the slope coefficient of food insecurity variable is found to be negative and statistically significant at a level of significance lower than 1%. This result reports that workers who experience moderate to severe food insecurity have a mean score of physical health that is 6.0370 [SD = 1.09; 95% CI = -8.19 to -3.88] points lower than the workers who have perfect food security or mild food insecurity. Besides having food insecurity, those who are somewhat happy with their workplace have 2.3423 [SD = 1.39; 95% CI = -5.09 to 0.40] units lower mean score, and those who are unhappy with their workplace have 3.6209 [SD = 1.77; 95% CI = -7.10 to -0.14] units lower mean score in their physical health status. The results of this study also reveal that workers who face health risks at the work have 2.7282 [SD = 1.15; 95% CI = -5.00 to -0.45] units lower mean score in their physical health status compared to their counterpart. However, the effects of marital status, education, work hours, monthly expenditure, and having a major disease on physical health are found to be statistically insignificant in this study.

The outcomes for mental health components are presented in Table 4. According to the outcome of this study, participants' mental health scores increase with age. Comparing to those workers who are in the 18–30 year age group, workers in the 41–50 year age group have a higher mean score of mental health by 2.5476 [SD = 1.45; 95% CI = -0.32 to 5.42] units, and workers in the age group greater than 51 years have a higher mean score by 3.0691 [SD = 1.55; 95% CI = 0.03 to 6.11] units. At a 10% level of significance, the slope coefficient of family size is revealed to be statistically significant and positive. It implies that workers with a family size of more than four have a mean mental health score that is 1.4474 [SD = 0.83; 95% CI = -0.19 to 3.08] units higher than the workers with a family size of four or fewer. Result also implies that, mean mental health score is 3.2418 [SD = 1.38; 95% CI = 0.52 to 5.97] units higher (on average) for families with monthly family expenditure exceeding BDT 30,000. When controlling for all other variables, it is found that there is a statistically significant and detrimental effect of food insecurity on mental health. Those who reported moderate to severe food insecurity on average had a lower mean score in mental health status by 3.5794 [SD = 1.03; 95% CI = -5.60 to -1.56] units.

**Table 4:** Result of Multivariate linear regression for mental health components (MCS-12)

Variables	Coefficient	Std. Err	*p*-value	95% CI	
**Age**	Ref. 18–30 Years				
31–40	-0.4954	1.0920	0.651	-2.6463	1.6555
41–50	2.5476^c^	1.4598	0.082	-0.3274	5.4232
≥ 51	3.0691^b^	1.5451	0.048	-0.0257	6.1126
**Gender**	Ref. Male				
Female	1.2255	1.2655	0.334	-1.2672	3.7182
**Marital Status**	Ref. Unmarried				
Married	0.4349	1.0024	0.665	-1.5394	2.4092
**Education**	Ref. No education				
Primary	-0.3241	1.2274	0.792	-2.7417	2.0935
Secondary	-0.3808	1.3036	0.770	-2.9484	2.1868
≥ Higher secondary	3.1381	2.4148	0.195	-1.6181	7.8944
**Family Size**	Ref. ≤ 4				
> 4	1.4474^a^	0.8323	0.083	-0.1919	3.0867
**Monthly expenditure**	Ref. ≤ 15,000 BDT				
15,001–30,000	-0.7046	0.8969	0.433	-2.4712	1.0619
> 30,000	3.2418 ^b^	1.3827	0.020	0.5182	5.9654
**Working hours**	Ref. < 8 h				
8 h	0.9367	2.5331	0.712	-4.0527	5.9262
> 8 h	-0.3428	2.2991	0.882	-4.8711	4.1855
**Moderate to severe food insecurity**	Ref. No				
Yes	-3.5794 ^a^	1.0262	0.001	-5.6007	-1.5581
**Satisfaction in workplace**	Ref. Happy				
Moderately happy	-3.8684 ^a^	1.3072	0.003	-6.4433	-1.2934
Not happy	-5.6789 ^a^	1.6569	0.001	-8.9424	-2.4154
**Health risk at workplace**	Ref. No				
Yes	0.8037	1.0828	0.459	-1.3291	2.9365
**Major Diseases**	Ref. No				
One	-4.7755 ^a^	1.0865	< 0.001	-6.9155	-2.6356
More than one	-8.3633 ^a^	0.9554	< 0.001	-10.2452	-6.4813
**Diagnostic check**					
R-Square	0.5085				
RMSE	5.8476				
F-Stat	13.3938 (*p*-value < 0.001)				

^a^ represents significance at 1% level, ^b^ represents significance at 5% level and ^c^ represents significance at 10% level

In this study, the coefficient of workplace satisfaction was found to have a negative sign with statistical significance. This result implies that, those who are somewhat happy with their workplace have a 3.8684 [SD = 1.31; 95% CI = -6.44 to -1.29] unit lower mean score, and those who are unhappy with their workplace have a 5.6789 [SD = 1.66; 95% CI = -8.94 to -2.42] unit lower mean score of mental health than those who are happy with their workplace. Finally, after adjusting for other variables, it is reported that having one or more serious illnesses has a statistically significant negative impact on the mental health of workers. In comparison to workers without a major disease, those with any one major disease have a lower mean score of 4.7755 [SD = 1.09; 95% CI = -6.92 to -2.64] units (on average), and those with more than one major disease have lower mean score of 8.3633 [SD = 0.96; 95% CI = -10.25 to -6.48] units (on average). In this study, the effects of gender, marital status, education, working hours, and workplace health risks on mental health are found to be statistically insignificant.

## Discussion

This study has explored food insecurity among workers from informally operated enterprises who belong to low-income and socially disadvantaged community. The empirical investigation of this study has found that workers experiencing moderate to severe food insecurity are more likely to face poor health status (physical and mental) than their food secure counterpart. This finding is congruent with past research that showed food insecurity to be associated with multiple health outcomes, e.g., poor sleep outcomes [18], cardio metabolic risk factors [63], hypertension [66], and cost-related underuse of medication [1]. Conversely, low-income working community with lack of job security can face financial hardships in managing their health conditions, which increases the subsequent risk of their food insecurity [36]. However, the cross-sectional nature of this study limits the understanding of the causal direction of the association, although both directions seem reasonable. The observed relationship between poor mental health status and severe to moderate food insecurity among workers in this study is supported by Jones [38]. The findings of this study are also in line with the studies of Brucker [14], Alvarez et al. [7], and Ziliak and Gundersen [79] that were conducted on adult populations with different characteristics. Besides, a few studies that were carried out in Bangladesh are also noteworthy in this context. Examining 176 informal waste workers in Bangladesh, Haque et al. [27] found that 81% of the participants went through psychological distress amid the COVID-19 pandemic. This study has also reported that during the pandemic many households were severely affected by a food crisis, which ultimately put them under mental strain with an increased risk of developing symptoms of poor mental health. The study by Rahman et al. [58] also revealed the deteriorating food insecurity statistics during the pandemic period which increased stress levels in Bangladesh. However, to the best of our knowledge, this is the first study to demonstrate an association between food insecurity and health status among low-income informal manufacturing workers, who are the severely affected section of the economy in the COVID-19 pandemic.

Aged workers (over 40 years) in the sample experience low mean score on the physical health component, implying that aging is negatively associated with workers’ physical health status. Older people are more likely to suffer from chronic diseases that require medication and more frequent medical visits. Low-income working adults hardly can afford that, and their physical health status deteriorates with age. However, it has been found that age positively correlates with workers' mental health. This result is in line with the research by Lau et al. [44] and Shamshirgaran et al. [68]. According to the findings of Lau et al. [44], older people had poorer physical health than those aged 18 to 34 years, but older people had better mental health related quality of life. Similarly, the findings of Shamshirgaran et al. [68] supported the notion that physical health deteriorated with age while mental health improved. Numerous empirical studies have supported the paradox of declining physical health but improving mental health with age, as was discussed by Thomas et al. [72]. Because older people are typically better at controlling their emotions and making complex social decisions, that improves mental health with age [12, 25]. Older people are also better able to cope with stressful changes [12, 37, 61]. Additionally, aging leads to an increase in wisdom and better mental health in later life [50, 78].

The study has found female workers to be more susceptible to weak physical health conditions. Informal women workers are more vulnerable with low incomes and high levels of food insecurity, increasing the risk for poor health, which is consistent with the finding of Horwood et al. [29]. However, Haque et al. [27] have found that female informal waste workers experience significantly more psychological distress than males in the context of Bangladesh. The study by Lau et al. [44] revealed that women scored worse on both physical and mental health. Family size of more than four members is strongly negatively associated with physical health, while it is weakly but positively associated with the mental health of workers. In low-income countries, early marriage, misconceptions about family planning, lack of access to health services, religious beliefs are connected to larger families and are often responsible for lowering health status. It is often seen in low-income families, in which parents live with hunger to feed their kids in days of extreme food crisis. However, in these social structures, children carry the family legacy and usually support their families’ livelihoods. Children may enter the labor force illegally and earn money for the family. In some cultures or communities, it is thought that the larger the family, the more blessed the family is. Besides, larger families may provide more opportunities for social support and caregiving, which can benefit mental health [10, 15, 71].

Monthly expenditures of more than BDT 30,000 can’t ensure physical health but can ensure mental health. The risk of developing mood and anxiety disorders is inversely correlated with disposable income and expenditure levels. Increased income and consequently increased expenditure may ease financial strain and give people a sense of security and control, which can act as a protective barrier against the damaging effects of stress on mental health as mentioned by Sareen et al. [64], Li et al. [46] and Thomson et al. [73]. Dissatisfaction with the work environment deteriorates both the physical and mental health related quality of life. Workers in the informal manufacturing sectors often report job dissatisfaction due to reasons such as low pay, working more than 8 h a day, job insecurity, unhealthy working conditions, abusive and exploitative environment. This finding is consistent with the finding of Fisher et al. [23] that reported job dissatisfaction with a strong correlation with symptoms of anxiety and depression. Besides, occupational health risks such as accidents while working or illness due to coming across poisonous chemicals contribute significantly and inversely to the physical health status of workers. Finally, the prevalence of major diseases is strongly and negatively related to workers mental health. This is in line with the findings of Alessi et al. [6] and Khandaker et al. [42] that stated any major disease such as heart disease and diabetes as increasing risk factor for mental health issues.

While empirical investigations have confirmed the objective of this study- association between food insecurity and health status- the results should be interpreted with caution given the limitations of this study concluded here. The first limitation is data constraint. The national datasets rarely provide information on the economic activities of micro businesses, especially of those that are not registered with the Industry and Commerce Department. While collecting primary data, the study faced movement restrictions and enterprise closure due to lock down and the pandemic context. This constraints the study to find a sufficient number of samples to make it more representative. The second limitation is the cross-sectional nature of this study, which precludes making causal inferences. The third limitation is that the predictor and outcome measures are based on self-report, which may be subject to response bias. The fourth limitation is the possibility of unmeasured confounders, although this study has tried to add a number of potential confounders, including age, sex, marital status, education, family size,, and health hazards related to the work place.

## Conclusions and policy suggestions

The present study adds insight to the existing literature on the nexus between food insecurity and the health status (both physical and mental) of working adults in informal settlements. The study uses a cross-sectional research method with primary data on workers from three categories of informal enterprises operating in three different areas of Dhaka city, Bangladesh. The food insecurity index is constructed from the collected data following FAO guidelines and SF12v2 mean scores are calculated to assess health status of informal workers based on self-reported PCS and MCS. The multivariate regression analysis has been applied, confounding with some socio-demographic and health-related factors, and the results show that moderate to severe food insecurity is a significant risk factor for the physical and mental health of informal sector workers. Moreover, age over 40 years, gender (female), a large family size, workplace dissatisfaction and health risks at work all negatively impact physical health status. Besides, age over 40 years, dissatisfaction with the work place, and the prevalence of one or more major diseases impact mental health status negatively, while a sufficient monthly expenditure positively impacts the mental health of the workers.

Given the overwhelming dominance of informal activities in developing countries, skills and productivity improvement of workers in the informal sector is a significant concern, for which ensuring a good health status is imperative. According to the ILO [33], pandemic circumstances require an immediate response to recovery, which cannot separate health from economic issues and to compensate for the loss of or reduction in economic activity, providing income and food support to individuals employed in informal settlements and to their families is the immediate policy response. Because food support for individuals in informal enterprises can ensure health and enhance productivity of workers by reducing their need to access health care services that are already limited for informal workers [33]. The outcome of this study has empirically confirmed these needs by establishing an inverse relation between food insecurity and health outcomes for informal sector workers in urban Bangladesh. This study expects to contribute to the argument in favor of the informal sector similar to the ILO. Such contribution will aid in containing the proliferation of pandemic-led economic meltdown as the informal sector is expected to incorporate strongly in post-pandemic economic restoration due to its potential to grow faster in periods of economic crisis with relative ease in creating new jobs (see [4, 48]). The outcome will also contribute to the Sustainable Development Goal 2 (SDG 2), where countries are committed to ending hunger and achieving food security by the year 2030 through the fulfillment of SDG target 2.1 (ensuring access to sufficient, safe, and nutritious food for all people, especially for the poor and people in vulnerable states all year round).

To address the urgent need to protect lives and livelihoods in a COVID-19 affected world, it is crucial to extend social and economic protection such as, universal health coverage and income support, to those most affected. This includes youth, women, older, and migrant workers in informal jobs who are poorly protected and low-paid [33]. Food insecurity issues among workers should be addressed carefully by the social community, local authorities, and enterprises. They should provide sufficient income and operate under a care system linking nutrition assistance and food support. Workers should take under routine health care practices. Enterprise-based food banks may aid in improving food insecurity among workers, particularly the elderly. Consumption support through transfers can also be helpful. It can be in cash if food markets are working and in kind, if they are not. The existing stigma of receiving food assistance might have increased mental stress and despair for those who were not food insecure before. This can be an argument for why food insecurity is associated with mental health. Therefore, measures should be taken to introduce food assistance as a social support system that may reduce the stigma and shame associated with accepting food support, either in cash or kind. Enterprise owners should come forward and address the physical and mental health status of workers carefully, as it is linked to productivity. The finding of this study- the identification of food insecurity as a determinant of health status from individual-level demographic and economic characteristics of a particularly vulnerable community- can assist the government and other organizations in targeting assistance and strategies. The findings of this study also emphasise on the need for randomized safety inspections in informal enterprises that can reduce injury rates, ensure health safety, and encourage a decent work environment. The formation of labor unions and their active role in the informal sector can boost the voice of workers, push for the prioritization of minimizing the formal-informal wage gap, and ensure health and safety issues in the workplace.

In the food availability category, Bangladesh scored the highest, 64.4 [24]. Still, it needs to develop its food supply and distribution adequacy by improving road, air, port, and rail infrastructures, modernization of food policy, corruption minimization, and political stability. Bangladesh should enhance the operation of the food safety net programs. In the post-pandemic economic context, effective measures and policies to save workers and bring back economic productivity must combine healthcare efforts with consumption support. Moreover, protecting workers’ rights, following workplace safety rules and other health practices, and ensuring access to decent work in all enterprises are decisive in addressing the health dimension of the ongoing crisis.

## Data Availability

The datasets generated and analyzed during the current study are not publicly available since it is a part of an ongoing research project. However, data can be available from the corresponding author on reasonable request with the consent of UniSQ.
